# Predicting Antidepressant Citalopram Treatment Response via Changes in Brain Functional Connectivity After Acute Intravenous Challenge

**DOI:** 10.3389/fncom.2020.554186

**Published:** 2020-10-06

**Authors:** Manfred Klöbl, Gregor Gryglewski, Lucas Rischka, Godber Mathis Godbersen, Jakob Unterholzner, Murray Bruce Reed, Paul Michenthaler, Thomas Vanicek, Edda Winkler-Pjrek, Andreas Hahn, Siegfried Kasper, Rupert Lanzenberger

**Affiliations:** Department of Psychiatry and Psychotherapy, Medical University of Vienna, Vienna, Austria

**Keywords:** treatment response prediction, major depressive disorder, selective serotonin reuptake inhibitors, functional connectivity, resting-state, functional magnetic resonance imaging

## Abstract

**Introduction:** The early and therapy-specific prediction of treatment success in major depressive disorder is of paramount importance due to high lifetime prevalence, and heterogeneity of response to standard medication and symptom expression. Hence, this study assessed the predictability of long-term antidepressant effects of escitalopram based on the short-term influence of citalopram on functional connectivity.

**Methods:** Twenty nine subjects suffering from major depression were scanned twice with resting-state functional magnetic resonance imaging under the influence of intravenous citalopram and placebo in a randomized, double-blinded cross-over fashion. Symptom factors were identified for the Hamilton depression rating scale (HAM-D) and Beck's depression inventory (BDI) taken before and after a median of seven weeks of escitalopram therapy. Predictors were calculated from whole-brain functional connectivity, fed into robust regression models, and cross-validated.

**Results:** Significant predictive power could be demonstrated for one HAM-D factor describing insomnia and the total score (*r* = 0.45–0.55). Remission and response could furthermore be predicted with an area under the receiver operating characteristic curve of 0.73 and 0.68, respectively. Functional regions with high influence on the predictor were located especially in the ventral attention, fronto-parietal, and default mode networks.

**Conclusion:** It was shown that medication-specific antidepressant symptom improvements can be predicted using functional connectivity measured during acute pharmacological challenge as an easily assessable imaging marker. The regions with high influence have previously been related to major depression as well as the response to selective serotonin reuptake inhibitors, corroborating the advantages of the current approach of focusing on treatment-specific symptom improvements.

## Introduction

Major depressive disorder (MDD) constitutes to one of the most prevalent psychiatric diseases and is seen as the second leading cause of disability worldwide (Spijker et al., 2004; Ferrari et al., 2013). Lifetime prevalence in industrialized countries are reaching almost 20% (Kessler and Bromet, 2013) and are even increasing (Weinberger et al., 2017). Moreover, ~30% of patients do not respond to standard medication and hence, suffer from depression longer than necessary before other therapeutic approaches are attempted (Souery et al., 1999). High prevalence and non-responder rates make MDD an obvious target for models of treatment success.

The ultimate goal is to find general predictive biomarkers that can be used to help make therapeutic decisions before determining the first intervention (Dunlop and Mayberg, 2014). During the last years, neuroimaging has steadily gained importance in this quest, with a distinct focus on changes in brain networks (Dichter et al., 2015) and processing of emotional stimuli caused by MDD (Langenecker et al., 2018a). Further promising results were also reported using, e.g., reward (Nguyen et al., 2019), emotional conflict (Fonzo et al., 2019) and response inhibition tasks (Tozzi et al., 2020). However, identification of neuroimaging markers or other treatment predictors is hampered by several factors: MDD is a highly heterogeneous disease, which is not only mirrored in the variety of symptom manifestations/trajectories (Drysdale et al., 2017; Hartmann et al., 2018) and neuroscientific findings but also the tools for diagnosis and assessment of severity vary considerably. Standard clinical questionnaires like the Hamilton depression rating scale [HAM-D; Hamilton (1960)] or Beck's depression inventory [BDI; Beck et al. (1961)] cover a broad range of symptoms but the overall depression severity is mostly seen as a mere sum of these.

Moreover, studies trying to identify treatment predictors often have to deal with small sample sizes that hardly allow for checking the generalizability on independent datasets. Hence, potential markers are frequently identified via mere correlation against quantified treatment responses. Due to considerable variability in neuroimaging data processing, correlations found can easily be specific for a given methodological setting [e.g., Borchardt et al. (2016)]. Moreover, findings also display specificity for the respective treatment under investigation (Brakowski et al., 2017). A further obstacle arises from the necessity that a predictive marker should be reliably measurable in the majority of patients. Within the fields of neuroimaging, resting-state (RS) functional magnetic resonance imaging (fMRI) constitutes one of the least demanding examinations, as it only requires subjects to lie still for a few minutes. Compared to task fMRI, it does not depend on performance, requires much less attention from the patients and is easier to standardize.

These characteristics have made RS fMRI a popular modality for investigating the effects of MDD on the brain's function and networks, and especially looking for markers aimed at a certain treatment response. Several studies claimed to have found predictors for various therapeutic approaches such as electroconvulsive therapy (Argyelan et al., 2016), transcranial magnetic stimulation (Avissar et al., 2017; Du et al., 2018; Weigand et al., 2018), psychotherapy (Crowther et al., 2015), mixed (He et al., 2016; Gong et al., 2018; Hou et al., 2018; Zhu et al., 2018), or single-product drug therapy (Alexopoulos et al., 2012; Fu et al., 2015; Cheng et al., 2017). Even though significant correlations with different RS functional connectivity (FC) metrics were found, out-of-sample validations or assessments of generalizability within the same sample [e.g., cross-validation (CV) or bootstrapping] were not reported in the publications above.

In order to address these challenges, the current work follows a multistage approach: Depressive symptoms within the sample were summarized according to latent sources using factor analysis (FA), which allows for investigation of effects of overall and symptom-specific severity. Using network based statistics [NBS; (Zalesky et al., 2010)], potentially predictable factors were identified by searching for related functionally connected brain networks. Finally, true predictive power was estimated using two validation schemes based on pseudo-independent data sampled from the study population and compared to analytically circular models built upon the previously identified networks, as they are sometimes encountered in the literature.

## Materials and Methods

This study was conducted in accordance with the Declaration of Helsinki, approved by the ethics committee and following the Good Scientific Practice guidelines of the Medical University of Vienna as part of a larger project registered at ClinicalTrials.gov (NCT02711215). Due to highly skew distributions, descriptive statistics are reported as “minimum/median/maximum” throughout this work.

### Subjects and Study Design

Thirty five patients suffering from MDD (19.65/26.96/55.28 years old, 16 females) were recruited in the outpatient clinic as well as at the hospital ward of the Department of Psychiatry and Psychotherapy of the Medical University of Vienna. Written informed consent was obtained from all participants. Two MRI examinations were conducted, 3/7/56 days apart, depending on the patients' availability. Inclusion criteria comprised of unipolar/first-episode MDD (according to DSM-IV, HAM-D ≥18) but otherwise general health (assessed via physical examination and the Structured Clinical Interview for DSM-IV) and no psychopharmacological treatment within the last three months, excluding the occasional intake of antihistamines and benzodiazepines. Subjects were excluded in case of former or current substance abuse, pregnancy and any MRI contraindications.

**Figure 1:**
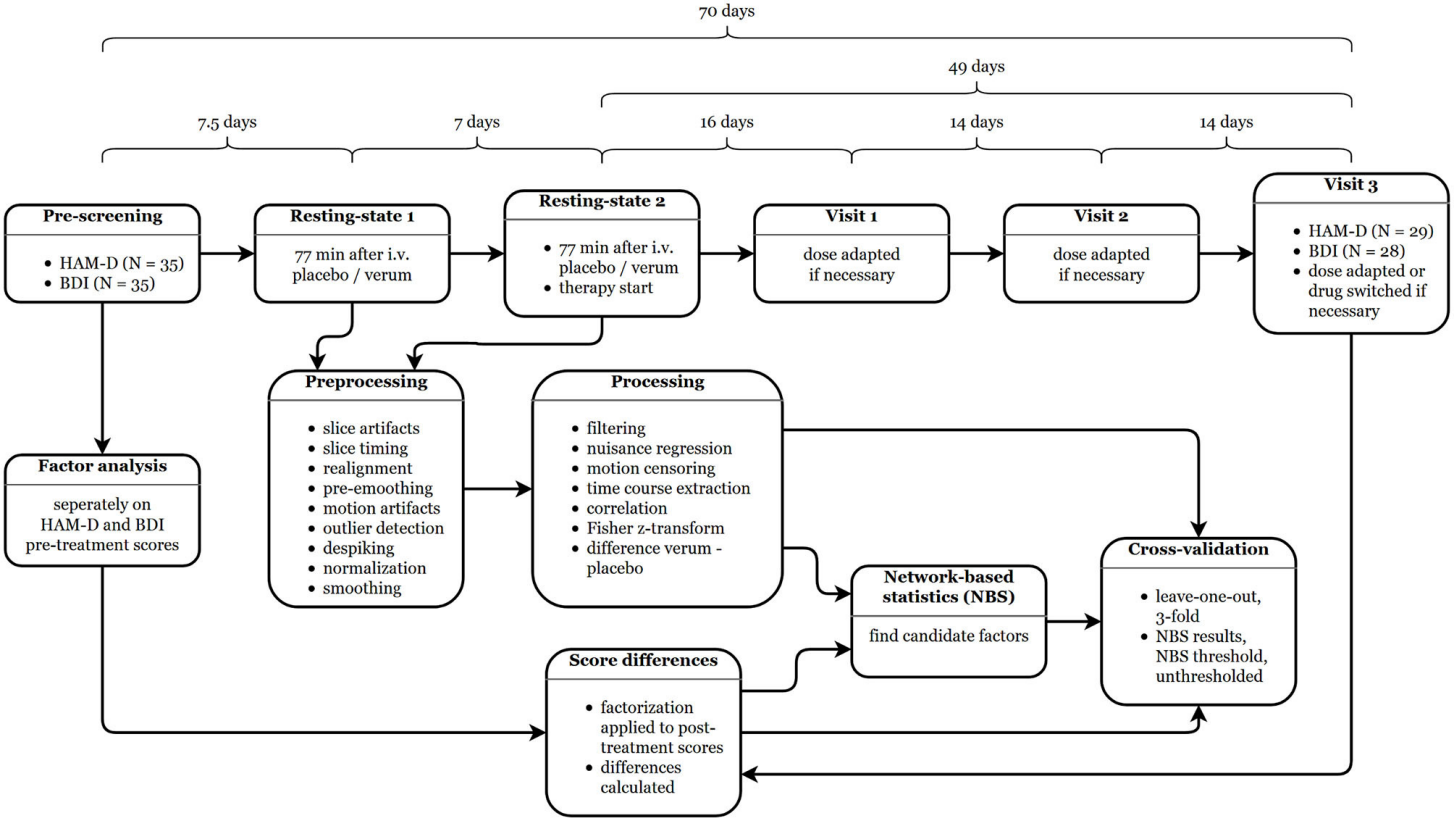
Study schedule and analysis workflow. The durations between the single steps are given as medians. HAM-D, Hamilton depression rating scale; BDI, Beck's depression inventory.

At each date, participants received either 8 mg of citalopram (a commonly used selective serotonin reuptake inhibitor—SSRI) diluted in 8 ml saline or a comparable amount of saline as placebo, injected intravenously (Kasper and Muller-Spahn, 2002) over 8 min in a randomized, double-blind, cross-over fashion (i.e., subjects receiving citalopram at the first scan received pure saline in the second one and vice versa). Successful drug application was ascertained by plasma levels drawn from arterial cannula placed in the left or right radial artery while the subjects were in the scanner (Alshelh et al., 2018). Starting the day after the second MRI examinations, patients received pharmacological treatment for MDD with an initial dose of 5 mg escitalopram (Cipralex—Lundbeck, Copenhagen, Denmark). Racemic citalopram was used for intravenous application since no syringeable escitalopram solution is currently available. In case of missing response (HAM-D decrease <50%), the dose was increased to an adequate level following therapeutic drug monitoring. This was done during visits carried out with ~2-week intervals, again depending on the patients' availability. All subjects received only escitalopram until the third visit and were switched to alternative medication (Duloxetin, Venlafaxin, or Mirtazapin) thereafter if necessary. For a detailed overview of the study schedule and analyses conducted see Figure 1.

### Psychometric Scoring

At each visit, depression severity was rated using HAM-D and BDI questionnaires, providing expert- and self-ratings due to known differences in the perception of symptom improvements (Lambert et al., 1986). Both questionnaires were previously reported to have high internal consistency and reliability (Bagby et al., 2004; Barkham et al., 2007). For the current analysis, only the assessment at inclusion (pre-scores) and at the last visit, where all subjects received escitalopram (post-scores; conducted 48/70/153 days after enrollment and 41/49/83 days after initiation of treatment), were used. In the end, the according psychometric data was available in 29 patients for HAM-D and 28 for BDI. For two subjects, post-scores were missing and linearly interpolated from the visit before and after, rounded up (no change in medication).

### Factor Analysis of Depression Scores

Given the number of existing but inconclusive factorization approaches for HAM-D and BDI, a FA appropriate for the current population was conducted and afterwards compared to previous results. Factorization was performed using SPSS 22 (IBM, Armonk, New York) on pre-scores only. Post-scores were automatically calculated from loading matrices to avoid influences of treatment response. For optimizing factors, scores with the lowest measure of sampling adequacy (MSA) were removed and FA repeated until the Kaiser-Meyer-Olkin (KMO) criterion was >0.5 (Backhaus, 2003) and the estimation converged. The following settings were used in SPSS: Factors were extracted using principal axis factorization, where the number of factors was determined via the Kaiser criterion (Guttman, 1954; Kaiser and Dickman, 1959), the analysis was based on correlation matrices, factors were orthogonally rotated using Varimax and the final scores were calculated employing the Anderson-Rubin approach. Varimax and the Anderson-Rubin method were used to enforce orthogonality of the factors and to ensure that the scores are uncorrelated (for details, see the SPSS manual).

### Resting-State Data Acquisition

Neuroimaging data was recorded using a 3 T Siemens Biograph mMR system (Siemens, Erlangen, Germany). The resting-state scans started 71/77/100 min after infusion of study medication (the time differences resulted from the acquisition of other sequences not presented here). In detail, 243 frames per run were acquired using the following parameters: repetition time 2.44 s, echo time 30 ms, 2.1 ×2.1 ×3 mm voxel size with 0.75 mm gap, 100 × 100 voxels in-plane, 36 slices, GRAPPA 2. Subjects were instructed to let their mind wander, look at a black crosshair on a gray background and stay awake.

### Resting-State Preprocessing

The RS data of the 29 subjects without missing HAM-D scores was preprocessed using MATLAB R2014a (The MathWorks, Natick, Massachusetts), Statistical Parametric Mapping, version 12 (SPM12^1^), and ArtRepair, version 5b (Mazaika et al., 2009)^2^. The following steps were performed: (i) correction of transient slice artifacts (ArtRepair), (ii) slice-timing correction (SPM), (iii) realignment (SPM), (iv), reslicing of realigned images (SPM), (v) pre-smoothing with 4 mm full width at half maximum (FWHM) to improve subsequent motion artifact correction (SPM), (vi) motion regression using the model by Grootoonk et al. (2000) (ArtRepair), (vii) detection of motion outliers (ArtRepair), (viii) despiking (ArtRepair), (ix) normalization to the standard space defined by the Montreal Neurological Institute (MNI) with an isotropic resolution of 2 mm (SPM).

### Resting-State Processing

Further RS-specific processing was conducted using in-house MATLAB code. Nuisance regression and frequency filtering were performed within one model as recommended by Hallquist et al. (2013). As nuisance parameters the first five principal components of white matter and cerebrospinal fluid were used, defined as the respective compartments of the Harvard-Oxford atlas^3^ thresholded at 95%. The passband was limited to 0.01–0.10 Hz. Frames identified as motion-related outliers using a modified version of the art_global MATLAB function (ArtRepair) with linear detrending to avoid misclassification of scanner drifts as movement were excluded from regression and further analysis. Mean time series were extracted from non-overlapping spheres of 10 mm diameter around the 264 region of interest centers defined by Power et al. (2011) (56/65/80 voxels), Pearson-correlated and Fisher-z-transformed given the parametric nature of the subsequent analyses. To reduce bias, only voxels present in all datasets were used for time course extraction.

### Network-Based Statistics Analysis

NBS (Zalesky et al., 2010) provide a way to tests connected networks (i.e., continuously linked nodes) with edges showing similar effects. The approach is based on connectivity matrices (functional or structural) and can be seen as an equivalent to the established cluster-level as compared to the voxelwise analysis on volumetric neuroimaging data. In the same manner, within the NBS framework, a general linear model is first estimated for each edge, using the change in the psychometric scores as regressor for the influence of intravenous citalopram on functional connectivity. Since this method allows for detection of widely distributed effects, it was utilized for a meaningful pre-selection of potentially predictable factors taking all connections into account simultaneously.

In detail, differences in connectivity z-matrices between the verum and placebo condition were used as input for NBS. The design matrices specified general linear models comprising of the respective score changes post-pre (HAM-D or BDI sum or factors), sex and age of patients, the mean connectivity averaged over all connections and conditions (Saad et al., 2013) and an intercept term. Connectivity thresholds were calculated as the *t*-value corresponding to *p* ≤ 0.001 at the available degrees of freedom. The significance threshold was set to *p* ≤ 0.10 to also cover trend-level effects (i.e., overall smaller networks). Extent (network size) and intensity (network strength) summary statistics were calculated for comparison as suggested by the NBS manual. For statistical inference, 10,000 permutations were performed. Results were not further corrected for the number of tests as this step was only conducted for selecting promising factors.

### Assessment of Generalizability

The degree of generalizability was estimated using CVs of complete and simplified models (without covariates) in order to mitigate overfitting. Weights for single connections were calculated as absolute value of the partial Pearson correlations (following the unidirectional NBS contrasts) with the respective coefficients being corrected for covariates and subsequently scaled by the standard deviation of all absolute correlations within the training set (models with signed weights were also calculated for comparison). The FC predictor for each subject was defined as the weighted average of difference of the z-matrices. Training set predictors, covariates and offset term were used afterwards to estimate linear regression models (with robust “bisquare” weighting in MATLAB) for the changes in the HAM-D and BDI factors, which were then applied to the test sets. Three selection procedures (NBS results, NBS threshold and direction of contrast, no threshold) and two resampling techniques (leave-one-out CV, LOOCV; 3-fold CV without role reversal of test and training sets, 1,000 redraws−3CV) were used to avoid assessment-specific results. Agreement between actual and predicted data was again estimated via Pearson correlation (offset and scaling are already accounted for in the models). The results were adjusted for the number of factors per score using the Sidak correction.

## Results

### Demographics

Six out of the 35 patients with depression enrolled in the study had to be excluded from model estimation due to missing post-scores and one for additionally missing BDI data. At inclusion, the remaining 29 subjects' HAM-D distribution was 18/21/38 and at the third visit 0/7/26. At this time, 16 subjects fulfilled the criteria for remission (*HAMD* ≤ 7) and 19 responded (HAM-D reduction of 50% or more). For the 28 subjects with both BDI scores, the respective values are 12/28/46 before and 0/16/38 after the investigated treatment period. The final datasets contained 14 female participants (13 for BDI models). No significant influences of the time between psychometric assessments or therapy duration on score reductions were found (Pearson and Spearman correlations all *p* ≥ 0.10).

### Factor Analysis

For HAM-D, the pre-scores of all 35 subjects were entered into the FA, 34 could be used for BDI (item enumerations see Table 1). After dropping HAM-D item 13, a KMO of 0.53 was reached and six factors explaining 48.50% of variance were identified using the Kaiser criterion. For BDI, a KMO of 0.52 was achieved after removing item T. However, since the procedure did not converge, item R with the next-lowest MSA was also removed, yielding a KMO of 0.56 and also six factors, which explain 57.48% of the overall variance. For easier understanding, the single factors are henceforth addressed according to the two items with the respective highest loadings (factor number in parentheses); HAM-D: late insomnia (1), intestinal-weight (2), agitation-insight (3), depressed-guilt (4), suicide-activities (5), insomnia-retardation (6); BDI: punishment-crying (1), self-negativity (2), pleasure-guilt (3), energy-sleep (4), irritability-concentration (5), sleep-sex (6).

**Table 1:** Factor analysis of Hamilton depression rating scale (HAM-D) and Beck's depression inventory (BDI) scores.

**HAM-D items**	**Factor number**						**BDI items**	**Factor number**					
	**1**	**2**	**3**	**4**	**5**	**6**		**1**	**2**	**3**	**4**	**5**	**6**
1	0.021	0.132	0.030	**0.576**	0.162	0.020	A	0.609	−0.026	0.438	0.458	0.036	0.181
2	−0.051	0.047	0.129	**0.608**	−0.270	0.100	B	0.175	0.206	0.358	0.110	−0.056	0.111
3	−0.174	0.172	0.265	−0.020	**0.684**	−0.049	C	0.374	0.674	0.249	0.103	−0.104	−0.050
4	0.209	0.323	−0.384	0.051	0.208	**0.424**	D	0.053	0.116	**0.773**	0.212	0.179	0.073
5	**0.820**	0.203	−0.118	−0.075	−0.147	0.034	E	0.375	0.391	**−0.511**	−0.054	0.251	0.031
6	**0.713**	0.018	0.136	0.101	0.074	0.130	F	**0.621**	0.249	−0.070	0.104	−0.008	−0.021
7	0.141	−0.007	−0.287	0.129	**0.442**	−0.003	G	0.098	**0.806**	0.183	0.367	−0.039	0.022
8	0.142	−0.171	0.253	0.031	−0.070	**0.653**	H	0.161	**0.738**	−0.037	−0.258	0.239	0.132
9	0.123	0.120	**0.462**	0.206	0.062	0.004	I	0.578	0.009	0.221	0.087	−0.028	−0.038
10	−0.127	0.019	0.258	0.359	−0.023	−0.420	J	**0.658**	0.229	−0.067	0.121	0.215	0.261
11	0.165	−0.015	−0.041	0.404	0.141	−0.197	K	0.446	0.272	0.203	−0.051	0.301	0.192
12	0.117	**0.821**	0.016	−0.028	0.255	−0.184	L	−0.02	0.298	−0.006	0.065	0.385	0.454
14	0.393	0.137	0.279	0.068	−0.019	0.099	M	0.249	0.168	0.180	0.563	−0.161	−0.215
15	0.173	0.321	0.295	−0.016	−0.341	−0.181	N	0.284	0.267	0.435	−0.126	0.116	0.248
16	0.154	**0.692**	0.091	0.292	−0.113	0.070	O	0.131	−0.01	0.142	**0.582**	0.330	0.072
17	0.065	−0.016	**0.796**	0.000	−0.036	0.071	P	−0.001	0.016	−0.042	**0.649**	−0.313	**0.586**
							Q	0.260	−0.145	0.379	0.286	**0.801**	0.068
							S	0.009	−0.056	0.054	0.079	**−0.435**	0.027
							U	0.135	−0.016	0.217	−0.032	−0.008	**0.670**

*The two highest-loading items are highlighted for each factor. Meaning of single items: 1: depressed mood, 2: feeling of guilt, 3: suicide, 4: insomnia: early in the night, 5: insomnia: middle of the night, 6: insomnia: early hours of the morning, 7: work and activities, 8: retardation, 9: agitation, 10: anxiety psychic, 11: anxiety somatic, 12: somatic symptoms gastro-intestinal, 13: general somatic symptoms (removed), 14: genital symptoms, 15: hypochondriasis, 16: loss of weight, 17: insight; A: sadness, B: pessimism, C: past failure, D: loss of pleasure, E: guilty feelings, F: punishment feelings, G: self-dislike, H: self-criticalness, I: suicidal thoughts or wishes, J: crying, K: agitation, L: loss of interest, M: indecisiveness, N: worthlessness, O: loss of energy, P: changes in sleeping pattern, Q: irritability, R: changes in appetite (removed), S: concentration difficulty, T: tiredness or fatigue (removed), U: loss of interest in sex*.

**Figure 2:**
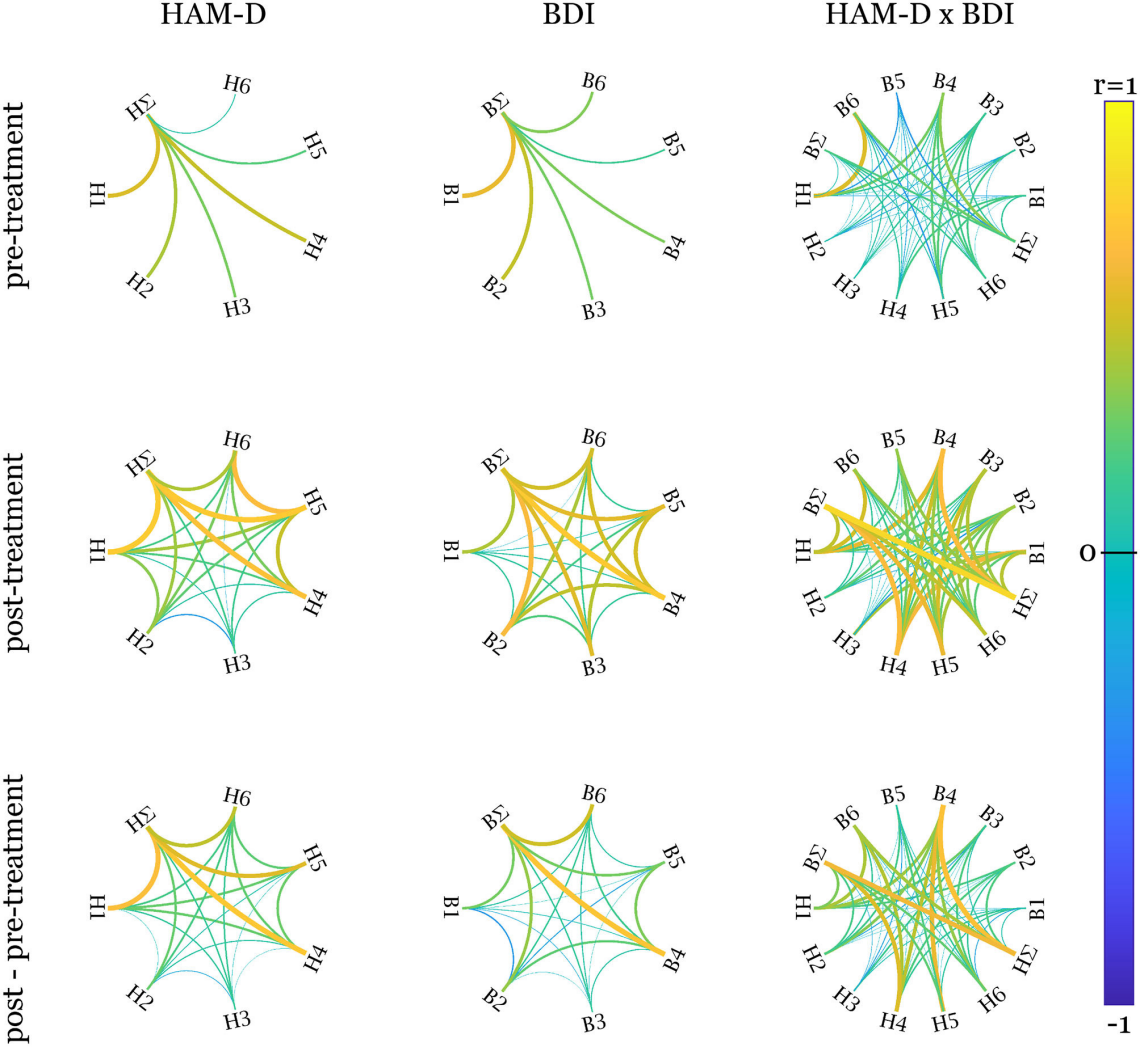
Pearson correlations within and between factors and sum scores. The left column displays the correlations within the factors of the Hamilton depression rating scale (HAM-D), the middle one of Beck's depression inventory (BDI) and the right one between HAM-D and BDI. The rows show the correlation graphs for pre-treatment, post-treatment, and difference scores. Since orthogonal factors were defined over pre-scores only to avoid influence of treatment, the corresponding correlations are 0. HAM-D factors: H1, late insomnia; H2, intestinal-weight; H3, agitation-insight; H4, depressed-guilt; H5, suicide-activities; H6, insomnia-retardation; BDI factors: B1, punishment-crying; B2, self-negativity; B3, pleasure-guilt; B4, energy-sleep; B5, irritability-concentration; B6, sleep-sex. ∑ indicates the sum scores. Created using Paul Kassebaum's circularGraph function (https://github.com/paul-kassebaum-mathworks/circularGraph).

The correlation structure of factor scores within and between the questionnaires is given in Figure 2. Within questionnaires, factor pre-scores are perfectly uncorrelated due to orthogonal Varimax rotation, only show occasionally higher correlations of post- but considerable relationships with sum scores. Between HAM-D and BDI, strong correlations of differences are found for “late insomnia” and “energy-sleep,” “depressed-guilt” and “energy-sleep”/“sleep-sex”/BDI sum, “suicide-activities” and “energy-sleep,” “insomnia-retardation” and “energy-sleep,” HAM-D sum and “energy-sleep”/“sleep-sex”/BDI sum. Sum post-scores also show a high correlation contrary to their pre-scores. Other relationships are especially pronounced between “insomnia” and “sleep” factors of HAM-D and BDI, respectively.

### Network-Based Statistics

**Figure 3:**
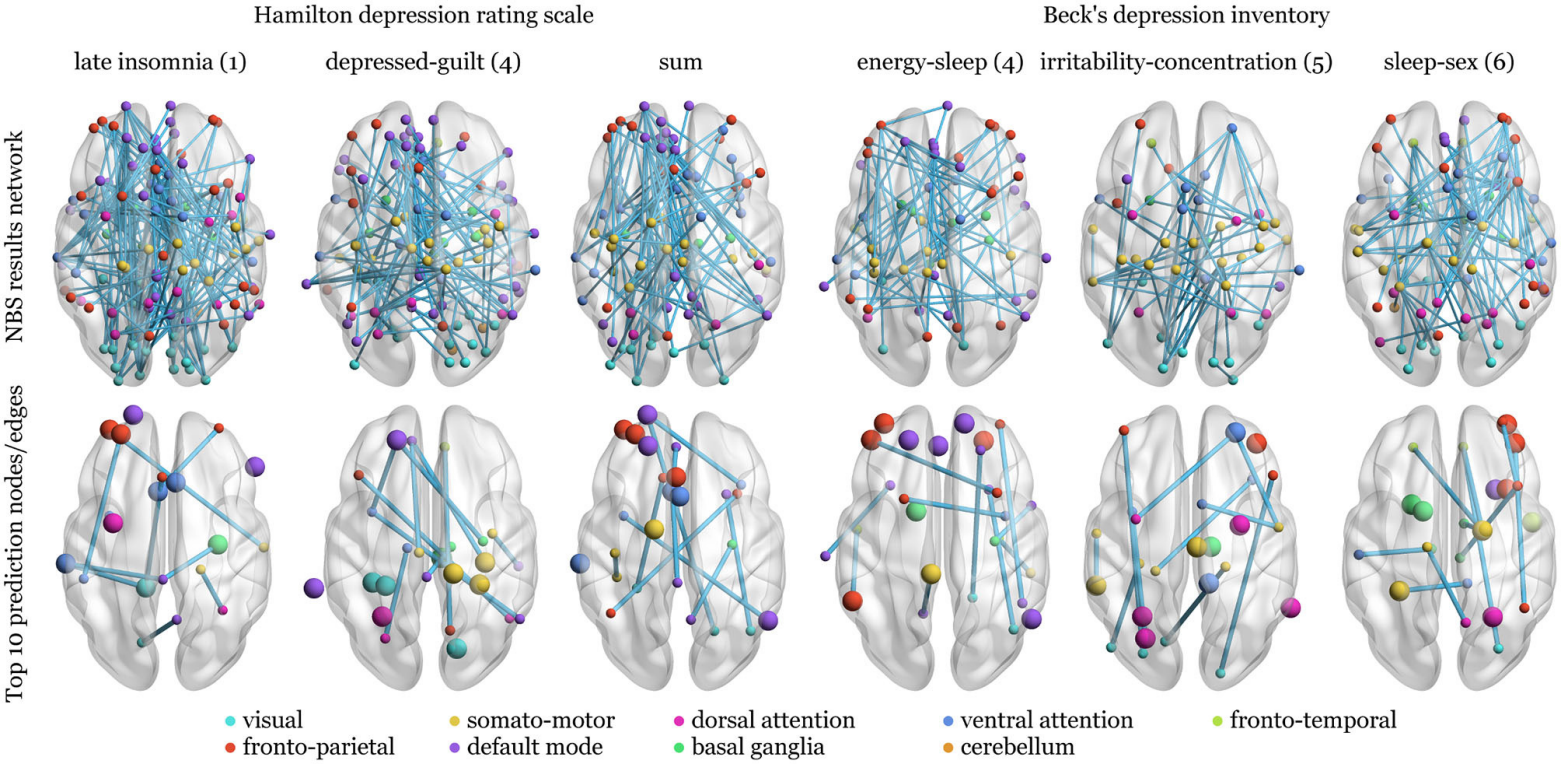
Network structure of the network-based statistics (NBS) selection of factors as prediction candidates and the top ten nodes and edges of the respective weight matrices for the models without application of any thresholds and 3-fold cross-validation. Upper row: Unweighted edges are shown since extent and intensity statistics led to the same networks. Differences in verum and placebo connectivity matrices were regressed against post—pre-treatment changes in Hamilton depression rating scale or Beck's depression inventory factors or sum, corrected for sex, age, and mean connectivity over conditions. The nodes are grouped according to the networks from Yeo et al. (2011) or anatomical region of the Harvard-Oxford atlas. Bottom row: The top ten nodes with the highest weight sums of adjacent edges (larger spheres) and single edges with the highest weights are depicted. Over all models, the top nodes are not necessarily connected to top edges (nodes without connections) and both are not necessarily included in the NBS results (top row).

Overall, 56 tests were conducted [2 scores *(6 *factors*+1 *sum*)*2contrast directions* 2 statistics]. No multiplicity correction was applied at this stage since the purpose of these tests was solely the preselection of potentially predictable candidate factors. Extent and intensity statistics were calculated for comparison and yielded the same results. Networks showing uncorrected or trend-level significance were found in 3 scenarios for HAM-D and BDI, respectively. Their structures are depicted in Figure 3. Score differences correlated negatively with the difference in connectivity between verum and placebo conditions for all reported cases except for the “irritability-concentration” and “sleep-sex” factors of BDI.

### Predictive Power of Citalopram-Induced FC Changes

Predictive power in terms of median Pearson correlation between predicted and actual data is presented in Table 2 for the full and reduced models. All models showed lower or at most equal predictive power with signed weights (results not shown).

**Table 2:** Median correlation coefficients as estimates of generalizability for models with and without covariates (sex, age, mean global connectivity).

	**Factor**	**leave-one-out cross-validation**			**3-fold cross-validation**		
		**NBS results**	**NBS threshold**	**no threshold**	**NBS results**	**NBS threshold**	**no threshold**
Models with covariates	HAM-D: late insomnia (1)	*r* = 0.94; *p* < 1E-13	*r* = 0.45; *p* < 0.05	*r* = 0.49; *p* < 0.05	*r* = 0.95; *p* < 1E-14	*r* = 0.51; *p* < 0.05	*r* = 0.55; *p* < 0.01
	HAM-D: depressed-guilt (4)	*r* = 0.83; *p* < 1E-7	*r* = 0.33; n.s.	*r* = 0.33; n.s.	*r* = 0.88; *p* < 1E-9	*r* = 0.40; n.s.	*r* = 0.40; n.s.
	HAM-D: sum	*r* = 0.90; *p* < 1E-10	*r* = 0.41; n.s.	*r* = 0.42; n.s.	*r* = 0.92; *p* < 1E-11	*r* = 0.51; *p* < 0.05	*r* = 0.51; *p* < 0.05
	BDI: energy-sleep (4)	*r* = 0.92; *p* < 1E-11	*r* = 0.07; n.s.	*r* = 0.19; n.s.	*r* = 0.94; *p* < 1E-12	*r* = 0.19; n.s.	*r* = 0.19; n.s.
	BDI: irritability-concentration (5)	*r* = 0.85; *p* < 1E-8	*r* = 0.24; n.s.	*r* = 0.21; n.s.	*r* = 0.88; *p* < 1E-9	*r* = 0.27; n.s.	*r* = 0.26; n.s.
	BDI: sleep-sex (6)	*r* = 0.88; *p* < 1E-9	*r* = 0.48; *p* < 0.05	*r* = 0.40; n.s.	*r* = 0.90; *p* < 1E-10	*r* = 0.41; n.s.	*r* = 0.40; n.s.
Models without covariates	HAM-D: late insomnia (1)	*r* = 0.83; *p* < 1E-7	*r* = 0.38; n.s.	*r* = 0.40; n.s.	*r* = 0.88; *p* < 1E-9	*r* = 0.47; *p* < 0.05	*r* = 0.48; *p* < 0.05
	HAM-D: depressed-guilt (4)	*r* = 0.67; *p* < 0.001	*r* = 0.00; n.s.	*r* = 0.23; n.s.	*r* = 0.79; *p* < 1E-6	*r* = 0.20; n.s.	*r* = 0.35; n.s.
	HAM-D: sum	*r* = 0.74; *p* < 0.001	*r* = 0.21; n.s.	*r* = 0.28; n.s.	*r* = 0.82; *p* < 1E-7	*r* = 0.35; n.s.	*r* = 0.40; n.s.
	BDI: energy-sleep (4)	*r* = 0.83; *p* < 1E-7	*r* = −0.07; n.s.	*r* = 0.08; n.s.	*r* = 0.88; *p* < 1E-8	*r* = 0.13; n.s.	*r* = 0.12; n.s.
	BDI: irritability-concentration (5)	*r* = 0.85; *p* < 1E-7	*r* = 0.26; n.s.	*r* = 0.24; n.s.	*r* = 0.89; *p* < 1E-9	*r* = 0.36; n.s.	*r* = 0.36; n.s.
	BDI: sleep-sex (6)	*r* = 0.71; *p* < 0.001	*r* = 0.29; n.s.	*r* = 0.03; n.s.	*r* = 0.78; *p* < 1E-5	*r* = 0.14; n.s.	*r* = −0.01; n.s.

*Absolute partial Pearson correlation coefficients were used for weighting. Naturally, estimations based on results from network-based statistics (NBS) show the highest agreement since the predictive matrix elements were selected using all of the data. Whether an additional threshold was used to exclude connections with low predictive potential does not make a substantial difference. P-values for the 1,000 runs of 3-fold cross-validation were calculated for n-2 degrees of freedom, where n is the number of subjects (assuming all subjects contributed to the result). HAM-D, Hamilton depression rating scale; BDI, Beck's depression inventory; n.s, not significant*.

**Figure 4:**
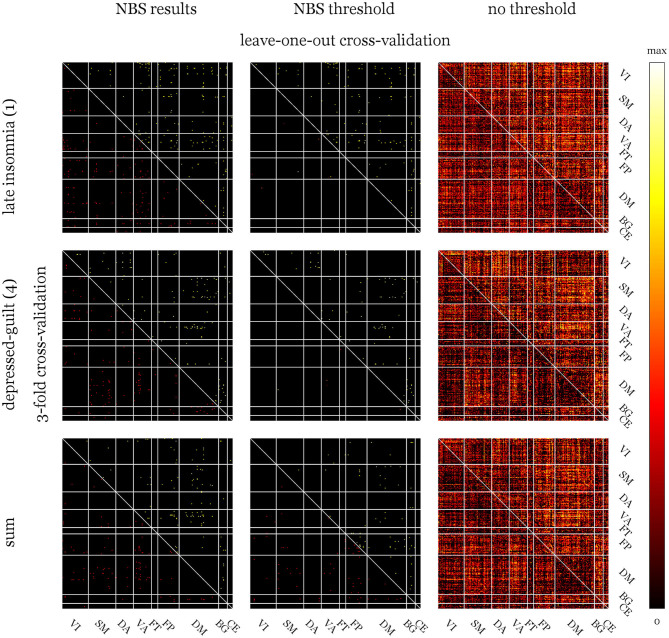
Median weight matrices for full models of derived Hamilton depression rating scale (HAM-D) factors and sum score using three selection strategies. Upper triangles show weights calculated via leave-one-out cross-validation (LOOCV) and lower ones for 3-fold cross validation (3CV) with 1,000 redraws. The left column shows rather equally distributed weights if validation is performed on the pre-selected edges of the network-based statistics (NBS) results. Using the NBS threshold (middle column) to eliminate connections with potentially low predictive capabilities, more of them could be considered in the 1,000 runs of 3CV compared to the number-of-subjects runs of LOOCV. This leads to 0 medians for most 3CV weights. If no threshold was used (right column), the patterns for both methods look quite similar. Weight colors represent the relative influence of the connections on the models and were scaled between 0 and the maximum of each matrix. Yeo atlas: VI, visual; SM, somato-motor; DA, dorsal attention; VA, ventral attention; FT, fronto-temporal; FP, fronto-parietal; DM, default mode; Harvard-Oxford: BG atlas, basal ganglia; CE, cerebellum.

**Figure 5:**
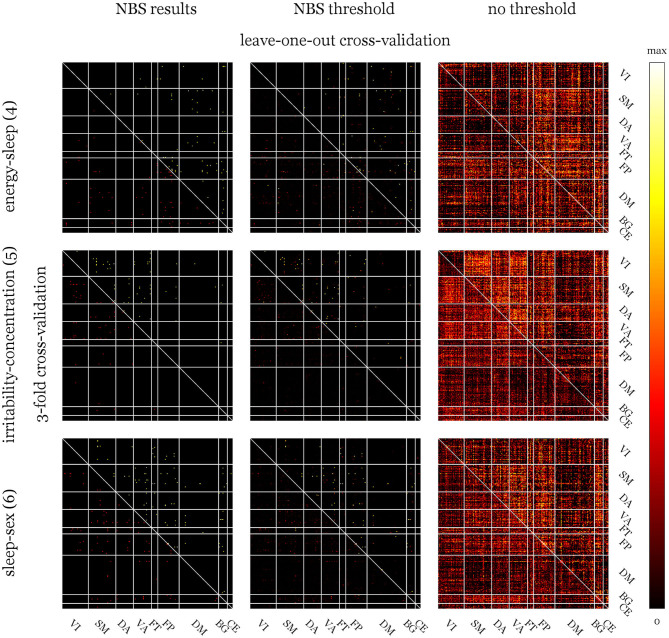
Median weight matrices for full models of derived Beck's depression inventory (BDI) factors. Abbreviations and explanations see Figure 4. Compared to the Hamilton depression rating scale (HAM-D), weight matrices for BDI show more pronounced patterns (especially for the analyses without threshold) indicating stronger influences of certain networks.

The highest correlation between actual and predicted scores is naturally achieved for connection differences, which were preselected based on the overall data. Being an obvious case of double-dipping, this estimation was only included for reasons of comparison. Whether a threshold was used to suppress connections with low predictive potential has no generally positive influence on the estimates. On the contrary, some predictions show higher agreement using all the connections. Correlations calculated for 3CV models are mostly larger than those for LOOCV indicating no influence of sample size but potentially influential subjects. Figures 4, 5 show the distribution of median weights for all tests on full models with regions of interest grouped according to Yeo et al. (2011) and basal ganglia and cerebellum regions from the Harvard-Oxford atlas. Due to the higher number of redraws and variance, median weights are smaller for 3CV with most of them being 0 if the NBS threshold is used. For the threshold-free models with covariates and evaluated using 3CV, the ten nodes and edges with highest sum and individual weights, respectively, are depicted in Figure 3. Predictors for HAM-D models feature especially high weights for the ventral attention (VA; e.g., anterior mid-cingulate cortex, left superior temporal and supramarginal gyrus, insula, eye fields), default mode (DM; e.g., frontal cortex, anterior and posterior cingulate cortex, precuneus) and fronto-parietal (FP; e.g., frontal and prefrontal cortex, anterior cingulate cortex) networks. For the BDI models, the FP (mainly mid-frontal cortex, including orbital parts), somato-motor (SM, for the “energy-sleep” factor; e.g., frontal regions, angular gyrus), and DM (e.g., pre- and postcentral gyrus, paracentral lobule, Heschl's gyrus) networks display high weights. Nearly all correlations between real and predicted data are higher for the full models when compared to the reduced ones, excluding the “irritability-concentration” model. The BDI factor “sleep-sex” is not predictable when no covariates and no threshold are used (the correlation between the predicted and the real data approaches zero).

**Figure 6:**
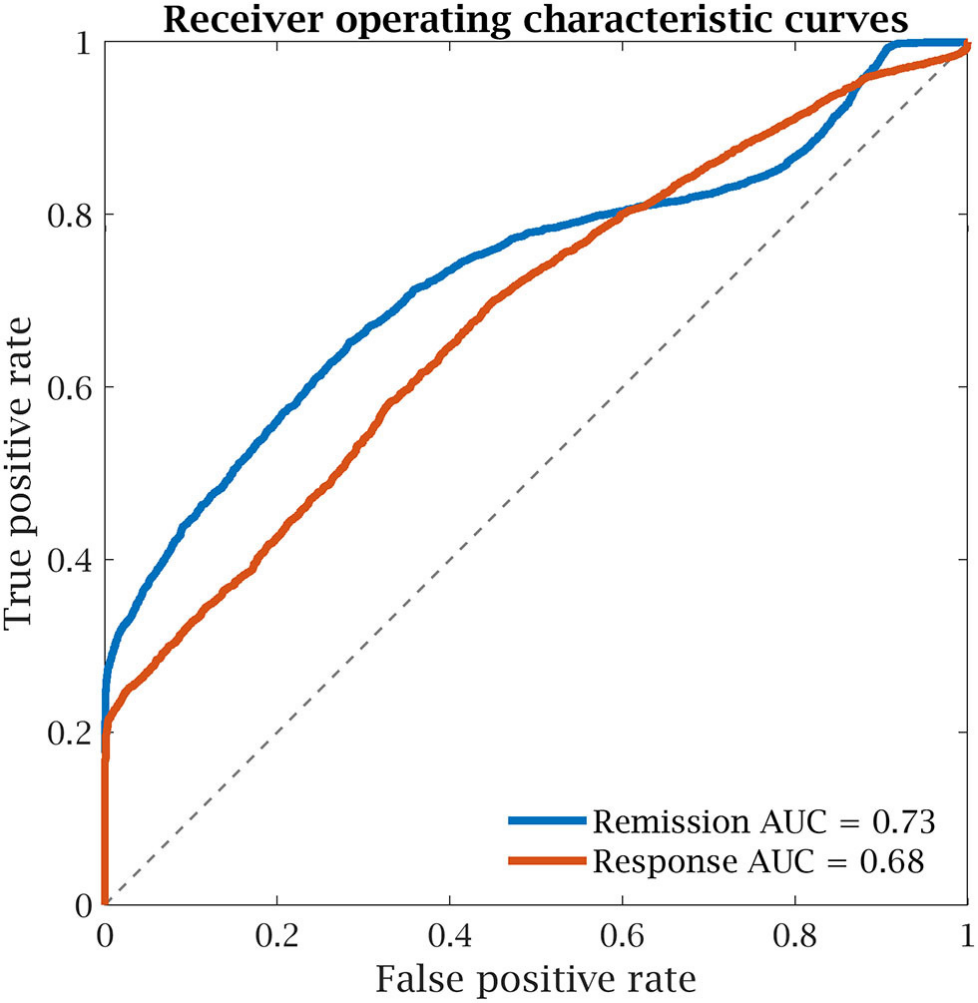
Receiver operating characteristic curves of the unthresholded model of the Hamilton depression rating scale (HAM-D) sum score with 3-fold cross-validation for remission (HAM-D ≤ 7) and response (HAM-D reduction ≥50%). AUC, area under the curve.

Since the model for the HAM-D sum score yielded significant results, it was further analyzed in terms of the area under the receiver operating characteristics curve (AUC) and balanced accuracy (BAC). For this, the usual clinical dichotomizations into remission and response were applied to the threshold-free predictions tested with 3CV and 1,000 redraws, as these represent the least restrained model: *AUC*_*rem*_ = 0.73, *AUC*_*resp*_ = 0.68, *BAC*_*rem*_ = 0.68 and *BAC*_*resp*_ = 0.60. The receiver operating characteristic curves of the *post-hoc* classification performance are presented in Figure 6.

## Discussion

The results indicate that short-term influences of citalopram on FC indeed contain predictive potential for certain clinician, but not self-ratings of antidepressant SSRI treatment response.

### Identification of Factors

An important advantage of FA is meaningful interpretability of results, which is much more difficult for other methods like principal, independent component, or canonical correlation analysis. The latter was also used in Drysdale et al. (2017) in the interest of identifying depression subtypes and FC correlates. Even though this approach yields linear combinations of symptoms that already correlate well with those of FC, additional data for independent validation is required. Moreover, in an attempt to replicate the findings in an independent sample, Dinga et al. (2019) also were not able to find similar structures, calling the generalizability of the study into question. Hence, a multistep approach was chosen for the current work keeping psychometric and RS data separated before creating prediction models.

Table 1 shows that negative influences on highest-loaded items are only found for two BDI factors (“guilty feelings” for the “pleasure-guilt” and “concentration difficulty” for the “irritability-concentration” factor). This opposing effect is indeed also observable in the original data: ρ = −0.32 , *p* = 0.07 for “loss of pleasure” and “guilty feelings” and ρ = −0.35, *p* < 0.05 for “irritability” and “concentration difficulty” (Spearman correlation, uncorrected). Several authors have performed exploratory [e.g., Brown et al. (2012), Lee et al. (2018)] and confirmatory [e.g., Morley et al. (2002), Tobias et al. (2017)] FA on BDI data of different patient populations with varying methods and results. In their work, Brown et al. (2012) report only positive correlations between the single BDI items in chronic fatigue patients using a two-factor model accounting for 35.7% of the overall variance. Lee et al. (2018) created a combined model of Beck's anxiety and depression inventories using a five-factor model (56.2% of combined variance explained) for a mixed group of psychiatric patients. Since those studies used either oblique rotation (allowing for correlation between factors, which was unwanted here) or did not further specify it, direct comparisons of the factors are difficult due to differently oriented coordinate systems.

As for BDI, HAM-D has also been subjected to FA for various scenarios. Broen et al. (2015) conducted a factorization in Parkinson's patients using principal component analysis, oblique rotation and the Kaiser criterion identifying six factors accounting for 59.2% of the total variance. Even though these numbers are very close to those reported here, comparisons beyond the magnitude of results are again impeded by different rotations. Also using principal component analysis and the Kaiser criterion, Olden et al. (2009) identified four latent HAM-D factors for patients suffering from terminal cancer. Since that study also used Varimax rotation, it can be easily derived via standardized regression coefficients that the “late insomnia” and “intestinal-weight” factors have likely equivalents in the “insomnia” and “somatic” factors, respectively (Table 3). A third “depression” factor might be partially represented in “suicide-activities” and “insomnia-retardation.”

**Table 3:** Comparison of the factors extracted from the Hamilton depression rating scale (HAM-D) by Olden et al. (2009) and the current analysis.

**Standardized β**	**late insomnia**	**intestinal-weight**	**agitation-insight**	**depressed-guilt**	**suicide-activities**	**insomnia-retardation**
Anxiety	−0.30	−0.51	−0.27	−0.07	−0.42	−0.69
Depression	−0.54	−0.16	−0.24	−0.06	0.28	0.27
Insomnia	0.74	−0.09	−0.27	0.06	0.16	0.05
Somatic	0.05	0.68	−0.29	0.12	0.13	−0.05

*The table shows regression coefficients β for z-scored columns modeling z-scored rows. “Insomnia” and “Somatic” seem to have a certain agreement with the “late insomnia” and “intestinal-weight” factors. “Depression” might be split up into “suicide-activities” and “insomnia-retardation,” where the negative influence of “late insomnia” could be connected to the strong representation of sleep items in two different factors. The exclusively negative coefficients for “Anxiety” are probably related to the comparably low loadings on the respective items (HAM-D items 10 and 11). This interpretation is also corroborated by the fact that the “depressed-guilt” factor shows the highest anxiety-item loadings and least negative β, whereas the “insomnia-retardation” factor behaves exactly the opposite way*.

The correlation structure of factors in Figure 2 indicates that not only the pre-treatment scores but also improvements in the single factors are, to a high degree, independent. Correlations in post-scores can be explained by general influences of medication on depressive symptoms. Factors with high loadings for similar items, especially the “insomnia” and “sleep” factors of HAM-D and BDI also display higher correlations emphasizing the appropriateness of their descriptions. Contrary to what could be expected and is reported in Kobak et al. (1990), Reynolds and Kobak (1995), this finding is not generalizable to sum pre-scores. Correlations of HAM-D and BDI pre-scores are only *r* = 0.21 but *r* = 0.82 for post- and *r* = 0.63 for difference scores. However, this discrepancy regarding the pre-treatment assessment is in line with Lambert et al. (1986) and the reason for investigating both scores. Assuming the expert-ratings are more accurate, this might indicate a biased self-perception with decreasing magnitude over the course of antidepressant treatment that lead to BDI scores being far less predictable from FC. From another perspective, the scores could also be seen as being based on different references: The clinician-based scoring might be influenced by having seen numerous patients suffering from differently severe depressions, whereas the self-rating is based on each patient's perception, which might be affected by a wide range of factors. Furthermore, these findings might also explain NBS results for BDI factors being trend-level significant at most. The partial similarity of weight distributions of the HAM-D models (see Figure 4) is also reflected in the respective correlations of the factors with the sum score.

### Predictive Power of Escitalopram Treatment Response Models

The overall aim of this work was to assess whether the response to antidepressant treatment (after a median of 10 weeks) with escitalopram can be predicted using short-term effects of citalopram on FC. To evaluate which aspects of depressive symptomatology are even represented in RS data, NBS analysis of drug influences was conducted. Three networks were identified for HAM-D (“late insomnia,” “depressed-guilt” factors and sum score) and BDI changes (“energy-sleep,” “irritability-concentration,” “sleep-sex” factors) with *p* ≤ 0.05 and *p* ≤ 0.10 uncorrected for the number of contrasts, respectively. Extent and intensity summary statistics yielded the same results (Figure 3). The networks themselves were of minor importance but afterwards used to demonstrate the effect of biased selection of predictors. Furthermore, the left and right columns of Figures 4, 5, as well as comparing the upper and lower row of Figure 3 indicate that many NBS connections were also assigned higher weights in the unrestricted case using all the connections. Predictive power expressed as Pearson correlation between estimated and real score reductions was at least twice as high (a factor of four in terms of explained variance) and approaching 1 when only edges of the NBS results were considered (Table 2). Since these were identified over all subjects, the scores that should be predicted already influenced the selection (“double dipping”).

Sikora et al. (2016) present a very similar ratio, where FC within the salience network (SN) explains 65% of the placebo response (*r* ≈ 0.81) but only shows a predictive power of *r* = 0.41. Concluding predictive capabilities from mere correlations is commonly encountered in literature even though CV or similar approaches could easily be employed to avoid overestimations and add a layer of generalizability.

For the subsequent models, reduced variants without covariates were evaluated to address overfitting (Table 2, lower half). These models performed worse for all but one (BDI: “irritability-concentration”) factor, suggesting that the additional information contained in these variables outweighs the increased model complexity. Hence, further investigations were limited to full models. However, the model for the BDI factor “sleep-sex” does not show any predictive capabilities when estimated solely on full correlation matrices, indicating that the result was driven by at least one covariate. Further analyses showed that correlations between predicted and real scores only approach zero when age is not included as a covariate. Indeed, subject age and change in “sleep-sex” factor significantly correlate (ρ = −0.48, *p* < 0.01, Spearman correlation), confirming this assumption. Still, in the preceding NBS analysis a trend-level significant network was found for the factor scores. This might either represent a false-positive result or the effect is only detectable in the corrective presence of an age covariate. However, there could be clinical relevance to this finding since it implies that improvements of depressive symptoms for this factor might be predictable to a certain degree from subject age alone. However, verification in independent samples is needed.

One HAM-D factor describing “late insomnia” and the sum score show significant correlations between predicted and real score differences after correction for multiplicity. Slightly higher agreements for 3CV compared to LOOCV could point toward influential samples. Since for LOOCV the model only changes by one subject between turns, influential ones will be present in the majority of estimates. 3CV, on the other hand, allows for more model variance as one third of the data is left out for each redraw. Robust “bisquare” weighting was used to mitigate the potential problem of leverage points, but residual effects might be present.

The finding that, besides the sum score, especially changes in a factor describing insomnia are predictable, could be of particular clinical interest. Some drugs with effects on serotonin reuptake/affinity to the serotonin transported are effective in the treatment of sleep disorders (Brietzke et al., 2019). Furthermore, insomnia is one of the possible side effects of escitalopram treatment (Burke, 2002; Waugh and Goa, 2003). Sleep disturbances at baseline themselves also carry predictive information for the antidepressant response (Manglick et al., 2013; Sung et al., 2015), which is partly reflected in the high correlations with the HAM-D sum in Figure 2 and the fact that the factors were defined based on the pre-treatment score alone. A stronger relationship between the post- and difference scores of the “sleep” factors (energy-sleep and sleep-sex) and the sum score can also be observed for the BDI. Furthermore, bidirectional relationships involving functional and structural connectivity between sleep disorders and MDD are known (Rosenberg et al., 2014; Khazaie et al., 2017). This all suggests a more complex relationship between SSRIs, sleep, and treatment response that demands further investigation. Furthermore, it seems that the predictability of changes in insomnia related to depression is either specific to FC or the influence of citalopram, since this factor could not be predicted in a well-powered study using structural and diffusion MRI (Yang et al., 2018).

Whether a priori thresholding of connections has any positive influence is not clear, but since each matrix element was weighted by the correlation with the regression target, which was also used to define the cutoff, the effect is probably small. When using multiple thresholded runs to build a final model, it needs to be noted that most single potential predictors are below the threshold most of the time (see the lower triangles of the median weight matrices in Figures 4, 5, middle columns). For sufficiently large datasets, algorithms that combine regularization, variable selection and prediction such as LASSO regression might provide a more powerful approach. However, in light of the sample size and the failed replication attempt of a promising model combining these steps (Drysdale et al., 2017; Dinga et al., 2019), they were kept separated.

### Influence of Functional Nodes and Networks

For the two full HAM-D models showing significant predictive power with or without thresholding, the estimated correlations range from *r* = 0.45 to 0.55–explaining ~20 to 30% of the variance. Sikora et al. (2016) found a relationship of similar strength (*r* = 0.41) between predicted and real responses to placebo but not antidepressant medication based on connectivity within the SN [included in the VA network of the Yeo atlas (Yeo et al., 2011)], especially the rostral anterior cingulate cortex. Figures 3, 4 show that connections within and including subregions of the anterior cingulate, which is known for being affected by MDD (Chen et al., 2018; Helm et al., 2018) as well as SSRIs (Arnone et al., 2018; Schrantee et al., 2018) contribute highly to the HAM-D predictors. A much stronger correlation between predicted and true absolute HAM-D scores of healthy and MDD subjects of *r* = 0.91 was reported by Qin et al. (2015). Amongst other regions, the posterior cingulate cortex, precuneus, insula, and basal ganglia were found to be mainly included in treatment-related connections. Those are also present in the nodes and edges with highest weights (Figure 3). Applied to the subset of clinically recovered patients, the model of Qin et al. (2015) failed, which is most likely caused by comparably greater variations between healthy subjects and patients. This assumption is supported by the fact that regions with therapy-related connectivity were identified as largely contributive across several of the current models.

A different approach was taken by Karim et al. (2018) for pharmacotherapy in late-life depression and Leaver et al. (2018) for electroconvulsive therapy applied to MDD. Patients were dichotomized into responders and non-responders allowing for classification via support vector machines. The first study used two emotional paradigms and RS fMRI at baseline, the same scans 1 day after a single dose of study medication and Montgomery-Asberg depression rating scale scores taken at baseline and 1 week into treatment. This setup achieved an AUC of 0.77 where important nodes for the support vector machine were largely located at the frontal gyri, including the orbital parts, at task and rest. These regions also display high individual prediction weights for all factors and might represent the influence of MDD on emotion processing (Loeffler et al., 2018). Furthermore, activation of these regions was shown to be indicative for future recurrence of depression (Langenecker et al., 2018b) and can be modulated using SSRIs (Wolf et al., 2018). Leaver et al. (2018) predicted electroconvulsive therapy outcomes with BACs of 58–68%: Across CV splits, FP, motor and superior temporal regions were most often selected as important nodes. The FP network indeed seems to play a universally pivotal role for predicting antidepressant response, whereas the SM network might be of especial importance for certain factors (Figure 3).

Komulainen et al. (2018) recently reported influences of escitalopram on FP regions in depressed patients after 1 week and before the onset of symptom improvements. Considering the current results, it might be possible that the FP network shows responses to SSRIs even within hours and speed or magnitude of this effect are, at least partially predictive for the antidepressant response. The motor network is usually not of primary interest in patients with MDD. Still, there are studies showing alterations in MDD (Sarkheil et al., 2020) and also predictive power of SM activation or connectivity for the response to serotonergic medication in Parkinson's (Ye et al., 2016) and MDD (Klimes-Dougan et al., 2018). Dunlop et al. (2017) investigated the predictive power of subcallosal cingulate cortex connectivity for antidepressant treatment with escitalopram, duloxetine (a serotonin norepinephrine reuptake inhibitor) or cognitive behavioral therapy. Remission after 12 weeks of medication could be identified with an AUC of 0.72 (no validation) based on the sum of subcallosal cingulate cortex FC to the ventromedial and ventrolateral prefrontal cortex/insula and midbrain. The AUC was higher for the sum of the FC scores compared to each one alone, corroborating the strategy of using a combination of nodes as predictor. Despite a certain consistency in influential connections, it must be noted that multivariate approaches do not directly allow for drawing conclusions on the importance of single features, i.e., one feature with high weight can easily be overruled by multiple others with lower weight. Even though dichotomization of outcomes was performed *post-hoc* in the current study, the results are of similar magnitudes (*AUC* = 0.73/0.68, *BAC* = 0.68/0.60) as those reported by Dunlop et al. (2017).

In a comparison of different machine learning algorithms, Patel et al. (2015) found that age, the mini-mental state examination and structural imaging features were predictive for the diagnosis of late-life depression, whereas for drug response, functional, and structural connectivity yielded the most generalizable results. Patel et al. (2015) also reports that support vector machines are outperformed by alternating decision trees but at least for structural data remain the prevailing machine learning approach for predicting antidepressant treatment response (Patel et al., 2016). This points toward a general underestimation of the predictive capabilities of neuroimaging for depression.

Other than the above-mentioned machine learning algorithms, the current study is not based on non-linear prediction but employs a simpler regression approach due to the moderate sample size. However, using a weighted mean to calculate a single predictor still allows for considering the whole brain at once. Another advantage of the current model is that a continuous outcome can be used avoiding arbitrary cutoffs and loss of statistical power (Kuss, 2013). Compared to studies using only one condition per patient, the influence of subjective traits, which might predict the probability of recovery rather than the response to a certain approach, can be excluded. This is especially important when RS fMRI is utilized where volunteers are usually instructed to let their mind wander. For instance, MDD patients with less pronounced lethargy might more think of physical activity leading to higher SM FC. Considering future studies, non-linearity can be taken into account in two ways: Firstly, the connectivity estimation itself can be based on methods other than the conventional linear Pearson correlation that, e.g., provide higher stability (Geerligs et al., 2016; Meszlényi et al., 2017). Secondly, more complex prediction algorithms can be used such as LASSO regression or the machine learning approaches discussed above. The linear methods used here can be seen as a special case of these approaches, which implies that they could only yield more accurate results given their requirements are met.

### Limitations

The main limitation that needs to be addressed is the available number of subjects. Starting at FA, there seems to be no agreement on sample sizes necessary to achieve stable results (Fabrigar et al., 1999; Henson and Roberts, 2006). However, it was shown that respecting quality criteria such as KMO and MSA in combination with a small number of factors already leads to reasonable outcomes (Preacher and MacCallum, 2002; Costello and Osborne, 2005) and FA can be reliably applied to small sample sizes (De Winter et al., 2009). Within the context of factor optimization, it was also necessary to remove items. Even though this is a common procedure, the respective items vary considerably between studies (Cole et al., 2004). Since no syringeable escitalopram solution exists, the racemic mixture was applied, possibly leading to a less pronounced effect on FC. The relationship between short- and long-term effects of citalopram might also be biased by additional influences on changes in depressive symptoms. These cannot be ruled out since placebo control during the treatment period was not possible. Another source of variation is the large range of time between scans and between psychometric assessments. This was not corrected for due to the already high number of predictors compared to samples but showed no significant relationship and any influence is also reduced by the resampling schemes employed. A higher sample size would also allow for selection of covariates within the model estimation process without considerably compromising stability (Heinze et al., 2018). Future studies should also explicitly estimate the influence of already collected psychometric data, which was out of scope of the current analysis but could constitute an individual baseline with additional measures steering the direction and magnitude of change (Karim et al., 2018).

### Conclusion

Using the short-term influence of citalopram on FC compared to placebo (saline), it was possible to predict improvements on one factor and the sum of the HAM-D at *r* = 0.45 to 0.55, validated using pseudo-independent data. The sum score model also performed reasonably well after *post-hoc* dichotomization into remitters and non-remitters (*AUC* = 0.73). Thus, based on the baseline depression severity assessed via the HAM-D and the individual improvements estimated by the model, the score after 7 weeks of escitalopram therapy can be predicted. This constitutes a clear advantage of the regression approach compared to predicting remission or response alone. The same approach, however, failed for BDI, likely due to a mismatch between self-perception of patients and expert perception of clinical symptoms. Including age, sex and a correction for the individual average connectivity was shown to increase predictive power of the models. In spite of in-sample testing, validation of results on independent datasets is needed. Furthermore, prior to an application of the model, a clinical validation is recommended, ideally involving a control group. Since also the HAM-D sum score was predictable, future studies could also take simpler approaches and concentrate on other methodological aspects to further increase predictive power. Additional studies are also needed in light of the different classes of antidepressant medication especially with promising alternatives such as ketamine and psilocybin (Witkin et al., 2018).

## Data Availability Statement

The processed data supporting the conclusions of this article will be made available by the authors, without undue reservation, to any qualified researcher.

## Ethics Statement

This study involving human participants was reviewed and approved by the ethics committee of the Medical University of Vienna. The patients/participants provided their written informed consent to participate in this study.

## Author Contributions

The data analysis was conducted by MK with assistance of MR and planned by AH and MK. The overall study was designed by GG, AH, EW-P, RL, and SK and also coordinated by GG. Neuroimaging data was recorded by LR and MK and quality control performed by MR and MK. Medical assistance and patient monitoring were provided by GMG, JU, PM, TV, and GG. Psychometric scoring was performed and validated by the same people. Medical supervision was provided by SK and EW-P. RL is the principal investigator and supervisor of the study. All authors have read and revised the manuscript and agreed to the final version.

## Conflict of Interest

Without any relevance to this work, SK received grants/research support, consulting fees and/or honoraria within the last 3 years from Angelini, AOP Orphan Pharmaceuticals AG, Celegne GmbH, Eli Lilly, Janssen-Cilag Pharma GmbH, KRKA-Pharma, Lundbeck A/S, Mundipharma, Neuraxpharm, Pfizer, Sanofi, Schwabe, Servier, Shire, Sumitomo Dainippon Pharma Co. Ltd., and Takeda. RL received travel grants and/or conference speaker honoraria within the last 3 years from Bruker BioSpin MR, Heel, and support from Siemens Healthcare regarding clinical research using PET/MR. He is shareholder of BM Health GmbH since 2019. TV received travel grants and compensation for workshop participation from Pfizer and Eli Lilly and speaker honorary from Shire. Preliminary findings of this study were presented at the workshop of the 32nd ECNP congress in Nice, 2019. Further analyses based on these data were presented at the annual meeting of the Organization for Human Brain Mapping, 2019, in Rome. The remaining authors declare that the research was conducted in the absence of any commercial or financial relationships that could be construed as a potential conflict of interest.

## Footnotes

^1^www.fil.ion.ucl.ac.uk/spm/software/spm12/

^2^cibsr.stanford.edu/tools/human-brain-project/artrepair-software.html

^3^fsl.fmrib.ox.ac.uk/fsl/fslwiki
